# Motion-corrected free-breathing LGE delivers high quality imaging and reduces scan time by half: an independent validation study

**DOI:** 10.1007/s10554-019-01620-x

**Published:** 2019-05-18

**Authors:** Gabriella Captur, Ilaria Lobascio, Yang Ye, Veronica Culotta, Redha Boubertakh, Hui Xue, Peter Kellman, James C. Moon

**Affiliations:** 1 Institute of Cardiovascular Science, University College London, Gower Street, London, UK; 2 Barts Heart Centre, The Cardiovascular Magnetic Resonance Imaging Unit, St Bartholomew’s Hospital, West Smithfield, London, UK; 3 Department of Cardiology, Sir Run Run Shaw Hospital, College of Medicine, Zhejiang University, No. 3 Qingchun East Road, Hangzhou, 310016 Zhejiang People’s Republic of China; 4 Cardiovascular Biomedical Research Unit, Barts and the London School of Medicine and Dentistry, Queen Mary University of London, London, UK; 5 National Heart, Lung, and Blood Institute, National Institutes of Health, DHHS, Bethesda, MD USA

**Keywords:** Late gadolinium enhancement, Image quality, Cardiac imaging, Fibrosis

## Abstract

Late gadolinium enhancement (LGE) cardiovascular magnetic resonance (CMR) sequences have evolved. Free-breathing motion-corrected (MOCO) LGE has potential advantages over breath-held (bh) LGE including minimal user input for the short axis (SAX) stack without breath-holds. It has previously been shown that MOCO-LGE delivers high image quality compared to bh-LGE. We sought to conduct an independent validation study to investigate real-world performance of bh-LGE versus MOCO-LGE in a high-throughput CMR center immediately after the introduction of the MOCO-LGE sequence and with elementary staff induction in its use. Four-hundred consecutive patients, referred for CMR and graded by clinical complexity, underwent CMR on either of two scanners (1.5 T, both Siemens) in a UK tertiary cardiac center. Scar imaging was by bh-LGE or MOCO-LGE (both with phase sensitive inversion recovery). Image quality, scan time, reader confidence and report reproducibility were compared between those scanned by bh-LGE versus MOCO-LGE. Readers had > 3 years CMR experience. Categorical variables were compared by χ^2^ or Fisher’s exact tests and continuous variables by unpaired Student’s t-test. Inter-rater agreement of LGE reports was by Cohen’s kappa. Image quality (low score = better) was better for MOCO-LGE (median, interquartile range [Q1–Q3]: 0 [0–0] vs. 2 [0–3], *P* < 0.0001). This persisted when just clinically complex patients were assessed (0 [0–1] vs. 2 [1–4] *P* < 0.0001). Readers were more confident in their MOCO-LGE rulings (*P* < 0.001) and reports more reproducible [bh-LGE vs. MOCO-LGE: kappa 0.76, confidence interval (CI) 0.7–0.9 vs. 0.82, CI 0.7–0.9]. MOCO-LGE significantly shortened LGE acquisition times compared to bh-LGE (for left ventricle SAX stack: 03:22 ± 01:14 vs 06:09 ± 01:47 min respectively, *P* < 0.0001). In a busy clinical service, immediately after its introduction and with elementary staff training, MOCO-LGE is demonstrably faster to bh-LGE, providing better images that are easier to interpret, even in the sickest of patients.

## Introduction

Late gadolinium enhancement (LGE) tissue characterization by cardiovascular magnetic resonance (CMR) has widespread applications so demand on image quality has grown [1, 2]. Breath-held (bh), inversion recovery (IR) electrocardiography (ECG)-gated, segmented spoiled gradient echo (GRE) readout was the gold standard sequence [3]. To improve speed and quality, new techniques emerged. These include phase sensitive IR (PSIR) [4], single shot LGE [5, 6], and motion corrected (MOCO) averaging with free-breathing [7, 8].

During traditional spoiled GRE bh-LGE sequences, a single image slice is acquired over a long breathhold (typically 12–16 heartbeats). If, instead of low flip angle spoiled GRE readout, steady-state free precession (SSFP) readouts are used, *k*-space can be acquired faster with increased signal-to-noise. This permitted single shot imaging and, for example, whole left ventricular (LV) coverage in a single bh [5]. However, trade off is needed so these approaches led to a combination of lower spatial resolution and longer read-outs with consequent reduced scar: remote myocardial contrast, even with PSIR [6]. One solution to improve this would be to use parallel imaging but it makes the images noisy. An alternative would be the development of single shot PSIR–SSFP LGE with parallel imaging and MOCO averaging, restoring both image resolution (matrix size) and signal-to-noise [1, 7, 8] with potential major advantages in clinical practice. Additionally, free-breathing CMR has advantages for patients, particularly the more unwell—it can potentially eliminate motion-related artifact and is faster to acquire [9] since pauses between bh slices are eliminated. Eliminating the need for bh voice commands brings benefits to patients with a language barrier, with hearing impairment, or to those than cannot stay awake. The strengths of MOCO-LGE were first described in a landmark clinical study 6 years ago [2], but in this time there have been no further replication studies and the promising research sequence, available only to selected centers, failed to mature to product sequence. We felt the need to remind the CMR community about the clinical utility, efficiency and easy-of-use of MOCO-LGE.

In consecutive patients referred for CMR in a high-throughput tertiary center, we sought to compare the clinical performance of a freshly introduced free-breathing single shot PSIR–SSFP with parallel imaging MOCO-LGE sequence against that of conventional bh segmented PSIR–fast low-angle shot (FLASH) LGE (bh-LGE).

## Methods

### Patient population

**Fig. 1:**
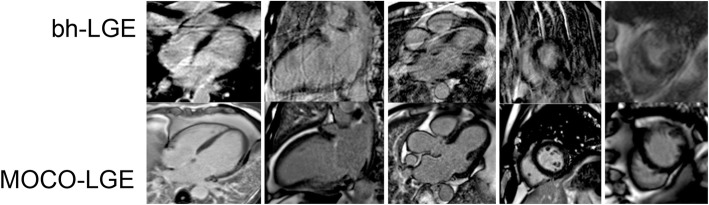
Example matched images from 11 selected patients who underwent both bh-LGE (top) and MOCO-LGE (bottom) showcasing the type of artifacts encountered. In the setting of arrhythmia or inability to breath hold, MOCO-LGE offers improved image quality. *bh-LGE* breath-held late gadolinium enhancement, *MOCO* motion correction

Four hundred consecutive consenting patients underwent CMR with either bh-LGE (n = 200) or MOCO-–LGE (n = 200), and a further 11 consenting patients underwent both (to permit Fig. 1). Patients were scanned on either of two 1.5 Tesla (T) magnets with standard contrast dose at the Barts Heart Centre, London, between July 2015 and December 2015. This period was immediately after the installation of the new MOCO-LGE sequence on scanners and followed elementary radiographer training in its use. We excluded patients with conventional contraindications to CMR and those with glomerular filtration rates < 30 mL/min. All participants provided written informed consent for imaging and clinical data to be used as part of the Barts Cardiovascular Registry. Clinical and comorbidity data were extracted from local electronic patient record systems. In-patient or out-patient status at the time of CMR was ascertained per patient. A composite score to represent clinical complexity (min 0–max 15) was estimated through the assignment of 1 point for each of the following clinically relevant variables if present: age ≥ 75 years , dementia, stroke, atrial fibrillation (AF), New York Heart Association functional classes III or IV, left ventricular ejection fraction < 35%, pericardial effusion, pleural effusion, ascites, severe anemia (hemoglobin < 8 g/dL), chronic kidney disease (glomerular filtration rate < 45 mL/min /1.73 m^2^ or creatinine > 200 mg/dL), in-patient status, high alcohol intake ( ≥ 14 units of alcohol /week), and recreational drug use.

### CMR protocol

All CMR scans were performed on one of two Magnetom Aera platforms (Siemens Medical Solutions, Erlangen, Germany) using an 18-channel phased-array anterior cardiac coil. The examination included standard bh segmented cine imaging with SSFP in the short axis (SAX) [10] (8 mm slice thickness with 2 mm gap) and 2-, 3-, and 4-chamber orientations. Real-time cines replaced segmented SSFP cines in patients with arrhythmias or breath-holding difficulties. LV volumes were measured as previously described [11]. The LGE protocol comprised separate 2-, 3-, and 4-chamber acquisitions, and an LV SAX stack (8 mm slice thickness with 2 mm gap) ensuring full LV coverage.

#### bh-LGE

Segmented PSIR–FLASH bh-LGE (standard Siemens Product) was performed 5–10 min after a 0.1 mmol/kg intravenous bolus of gadoteric acid (Dotarem; Guerbet, France). All reconstructions were with PSIR [4]. This sequence acquires IR and proton density (PD)-weighted data every other heartbeat (every third heartbeat for faster heart rates > 90 beats /min). Typical parameters were: an adiabatic 180° inversion pulse every second R-R, field of view (FOV) ~ 360 × 270 mm, acquisition matrix ~ 256 × 138, spatial resolution ~ 1.4 × 2.1 mm (8 mm slice thickness), repetition time/echo time (TR/TE) 8.2/3.17 ms, flip angle 23°, 23 lines per acquisition window, inversion time (TI) starting at ≈ 300 ms (adjusted for nulling non-infarcted myocardium), imaging window 189 ms, pixel bandwidth 140 Hz, no parallel image acceleration. Typical bhs were 14 heartbeats in duration.

#### MOCO-LGE

Single shot PSIR–SSFP free-breathing respiratory MOCO-LGE [8] (research sequence) was performed at the same post contrast delay and contrast dose and using similar typical TIs, slice thickness and FOV. The matrix and spatial resolution were however slightly higher 256 × 144 and 1.4 × 1.9 mm (8 mm slice thickness). Other parameters were TR/TE 2.8/1.18 ms, pixel bandwidth 1085 Hz, generalized autocalibrating partial parallel acquisition (GRAPPA) = 2, flip angle 50°. Each acquisition had eight repeated measurements per slice with each measurement every second R-R interval (every third for faster heart rates > 90 bpm) for a duration of 16 heartbeats (or 24 heartbeats for faster heart rates > 90 bpm). Non-rigid image registration corrected respiratory motion between repeated measurements [1, 7, 8]. The details can be found in reference [9]. The MOCO-LGE sequence is similar to the standard Siemens product (currently available on the Vida and Sola) but is implemented in the Gadgetron streaming reconstruction software framework [12] which provides on-the-fly reconstruction for increased speed. At the end of acquiring a SAX stack of slices, the complete reconstruction with MOCO averaging is completed in less than 10 s.

#### Image quality and reader confidence analysis

**Fig. 2:**
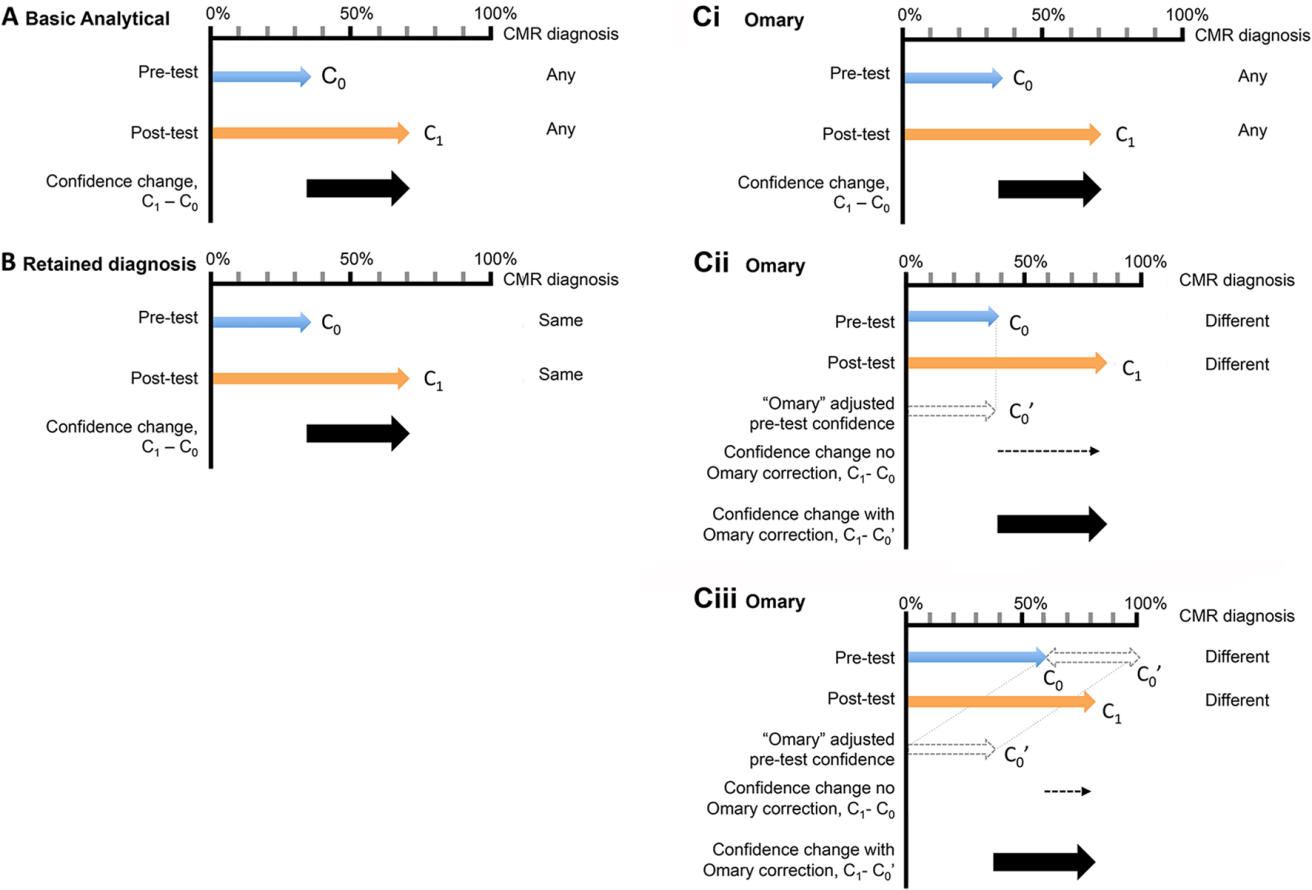
Reader confidence estimation methods used in this study. **a** Basic analytic method: C_0_ and C_1_ denote pre- and post-test confidence on a 0% to 100% scale, irrespective of whether the pre- and post-test diagnosis matched or not. **b** Retained diagnosis method: removes from consideration cases in which the post-test diagnosis differs from the pretest diagnosis and only considers reads with unchanged (or “retained”) diagnoses and **ci**–**iii** Omary method: considers all cases and estimates C_1_ minus C_0_ except for the situation where diagnoses differ and C_0_ is < 50%, in which case C_1_ is calculated as: C_1_ − (100 – C_0_)

Quality of bh-LGE and MOCO-LGE images was evaluated by Reader-1 (IL, cardiologist with > 3 years’ CMR experience), blinded—as far as possible—to LGE technique using cvi42 post-processing software (Version 5.1.1, Circle Cardiovascular Imaging, Inc., Calgary, Canada). We adapted an established quality scoring method [13] and assessed 10 criteria, of which the first 9 refer to the LV SAX LGE stack. Every criterion was scored from 0 (excellent) to 3 (worst) to obtain a final composite score (Table 1). The total minimum attainable score was 0 for perfect image quality, and the maximum score 31 for worst image quality. Incorrect TI was not included as both approaches offered PSIR reconstruction. Reader confidence in LGE diagnoses was measured using three well-established methods as previously described [14] and detailed in Fig. 2.

**Table 1:** Adapted qualitative scoring of LGE images (10 image quality criteria appraised)

LGE criterion	0	1	2	3	Max
LV coverage^a^	Full coverage	–	Apex not covered	Base or ≥ 1 slice missing	5
Wrap	No	1 Slice	2 Slices	≥ 3 Slices	3
Respiratory ghost (motion artefact)	No	1 Slice	2 Slices	≥ 3 Slices	3
Cardiac ghost (motion artefact)	No	1 Slice	2 Slices	≥ 3 Slices	3
Blurring/mis-trigger	No	1 Slice	2 Slices	≥ 3 Slices	3
Metallic artifacts	No	1 Slice	2 Slices	≥ 3 Slices	3
Signal loss (coil inactive)^b^	Activated	–	Not activated	–	2
Slice thickness^c^	≤ 10 mm	11–15 mm	–	> 15 mm	3
Inter-slice gap^c^	< 3 mm	3–4 mm	–	> 4 mm	3
Correct LV long axis^d^	≥ 3	2	1	None	3
Total LGE score					31

*LGE* late gadolinium enhancement, *LV* left ventricle

^a^For ‘LV coverage’, maximum (and worst) possible rating for this criterion was 5; inadequate apical coverage (2 points); inadequate basal coverage; ≥ 1 additional slice(s) missing (3 points)

^b^If relevant coils had not been activated resulting in signal loss, 2 points were given, otherwise 0

^c^Slice thickness and slice gap were fixed for our protocols so all study patients scored 0 for this criterion

^d^For ‘Correct long axis’ 3 points were given if all long axis slices were missing (4-, 3-, and 2-chamber), 2 points if 2 long axis images were missing, 1 point if 1 long axis was missing

#### Scan timings

Acquisition times for LGE imaging were semi-automatically derived from the digital imaging and communications in medicine (DICOM) time stamps on the first and last image of each module using an in-house Matlab (Mathworks, Natick, US) script.

#### LGE reporting

**Fig. 3:**
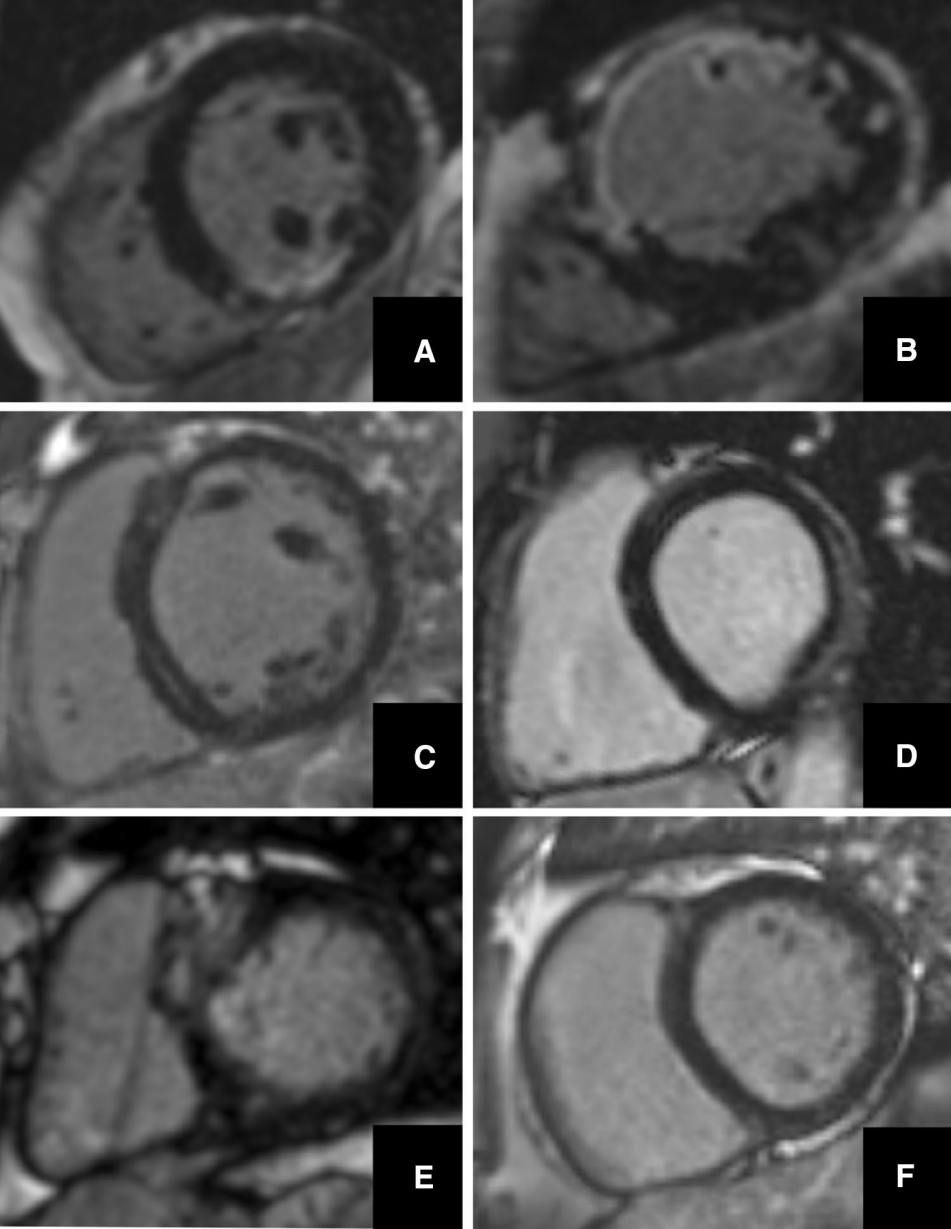
Example MOCO-LGE images illustrating the variety of LGE patterns observed in the sampled cohort. **a** Subendocardial chronic myocardial infarction, **b** transmural chronic myocardial infarction, **c** mid-wall enhancement in patient with dilated cardiomyopathy, **d** basal lateral wall subepicardial enhancement in a patient with previous myocarditis, **e** patchy anteroseptal scar in a patient with hypertrophic cardiomyopathy and **f** trace of superior and inferior right ventricular insertion points. Other abbreviations as in Fig. 1

Prior to commencing review of an LGE dataset, Reader-1 reviewed the CMR referral letter and clinical details in the electronic health record, and predicted the likelihood of finding pathological LGE as well as the pretest confidence in the ensuing LGE diagnosis (0–100%). Next, the LGE data was reported (as presence/absence of LGE ± pattern, Fig. 3) and a post-test confidence (0–100%) for this LGE ruling provided. To determine inter-rater reproducibility of LGE reports, the entire analysis (n = 400) was repeated by a blinded Reader-2 (VC) with equivalent CMR experience.

### Statistical analysis

Statistical analysis was performed in R (version 3.0.1, The R Foundation for Statistical Computing). Descriptive data are expressed as mean ± standard deviation except where otherwise stated. Distribution of data was assessed on histograms and using Shapiro–Wilk test. Categorical variables were compared using χ^2^ or Fisher’s exact tests. Continuous variables were compared using unpaired Student’s *t*-test. Inter-rater agreement of LGE reports was calculated using the Cohen’s kappa statistic. A two-sided *P* value < 0.05 was considered significant.

## Results

### Patient characteristics

Clinical and demographic characteristics of patients are summarized in Table 2. One-hundred and thirty three patients (33%) were clinically complex. Bh-LGE and MOCO-LGE populations were similar across most clinicodemographic characteristics including their burden of AF/flutter (16% vs 22%, *P* = 0.368), except that patients imaged by MOCO-LGE were slightly older, with lower ejection fractions, and more clinically complex (scores for bh-LGE and MOCO-LGE groups: 0.41 ± 0.84 vs 0.72 ± 1.15, respectively, *P* = 0.003).

**Table 2:** Clinical and demographic characteristics of study patients

Variable	MOCO-LGE (n = 200)	bh-LGE (n = 200)	*P* Value
Demographics			
Female (%)	85 (43)	72 (36)	0.219
Age (y)	55 ± 16	50 ± 15	**0.001**
Ethnicity			
White (%)	120 (60)	108 (54)	0.543
Black (%)	16 (8)	36 (18)	0.096
Mixed/multiple (%)	2 (1)	4 (2)	0.622
Asian, Asian British (%)	46 (23)	40 (20)	0.698
Other	16 (8)	12 (6)	0.698
Clinical characteristics			
Body mass index (kg/m^2^)	29 ± 6	28 ± 6	0.096
Diabetes mellitus (%)	50 (25)	40 (20)	0.434
Hypertension (%)	138 (69)	112 (56)	**0. 032**
Dyslipidaemia	102 (51)	82 (41)	0.120
Current cigarette smoking	102 (51)	70 (35)	0.113
History of atrial fibrillation or flutter	44 (22)	32 (16)	0.368
Inpatient status	20 (10)	12 (6)	0.183
Prior coronary revascularization	30 (15)	32 (16)	0.887
Acute myocardial infarction	4 (2)	8 (4)	0.503
Prior myocardial infarction	60 (30)	48 (24)	0.342
Clinical complexity score	0.72 ± 1.15	0.41 ± 0.84	**0.003**
Laboratory characteristics			
Creatinine (mg/dL)	84 ± 29	85 ± 31	0.739
Glomerular filtration rate (mL/min/1.73 m^2^)	86 ± 26	85 ± 25	0.695
Clinical indication for CMR			
Known or suspected cardiomyopathy	41 (21)	54 (27)	0.580
Possible CAD/stress perfusion	106 (53)	95 (48)	0.317
Myocarditis (new or follow up)	9 (5)	14 (7)	0.389
Evaluation for arrhythmia substrate	11 (6)	8 (4)	0.639
Family screening	5 (2)	3 (1)	0.723
Adult congenital heart disease	4 (2)	7 (3)	0.543
Mass or thrombus	3 (1)	2 (1)	1.000
Other	21 (10)	17 (9)	0.610

Data reported as mean ± standard deviation, counts (%) or median (interquartile ranges 1–3). Other abbreviations as in Table 1

Significant *P* values highlighted in bold

*bh* breath-held, *MI* myocardial infarction, *MOCO* motion-corrected, *CAD* coronary artery disease, *CMR* cardiovascular magnetic resonance, *y* years

### Global scan protocol evaluation

Eight (4%) and 6 (3%) patients from the bh-LGE and MOCO-LGE groups respectively received realtime cines corroborating the slightly higher complexity of the MOCO-LGE cohort. There were no other systematic differences in acquisition (e.g. the LV SAX cine stack acquisitions were similar: bh-LGE 03:47 ± 01:21 min vs. MOCO-LGE 03:58 ± 01:40 min, *P* = 0.227), Table 3).

**Table 3:** CMR characteristics of study cohorts

CMR Variable	MOCO-LGE (n = 200)	bh-LGE (n = 200)	*P* value
LV ejection fraction (%)	60 ± 13	63 ± 11	**0.013**
LV mass index (g/m^2^)	65 ± 30	63 ± 21	0.440
LV end-systolic volume index (mL/m^2^)	36 ± 26	32 ± 16	0.065
LV end-diastolic volume index (mL/m^2^)	83 ± 29	81 ± 20	0.423
Left atrial area (cm^2^)	12 ± 3	12 ± 3	1.000
LV wall thickness			
None or borderline (< 10 mm|0–13 mm)	174 (87)	172 (86)	0.764
Mild or moderate (14 mm|≥ 15 mm < 30 mm)	26 (13)	28 (14)	
Severe (≥ 30 mm)	0 (0)	0 (0)	–
Effusion (pericardial, pleural, ascites)	26 (13)	20 (10)	0.434
LGE data			
No LGE	119 (60)	120 (60)	1.000
LGE pattern			
Subendocardial chronic MI	27 (13)	24 (12)	0.764
Transmural chronic MI	21 (10)	15 (7)	0.383
Acute MI MVO dark core	1 (1)	1 (1)	1.000
Mid-wall	15 (8)	19 (10)	0.590
Subepicardial	14 (7)	15 (8)	1.000
Patchy	14 (7)	16 (8)	0.841
RV insertion points	25 (12)	21 (11)	0.639
LGE not analysable	0 (0)	1 (1)	1.000
Image quality score	0 (0–0)	2 (0–3)	** < 0.001**
Reader confidence by method			
Basic analytic	24.0 ± 16.2	15.9 ± 18.4	** < 0.0001**
Retained diagnostic	22.8 ± 15.4	15.9 ± 16.9	** < 0.0001**
Omary correction	32.2 ± 21.3	24. 5 ± 22.3	** < 0.0004**
LGE SAX stack module (min)	03:22 ± 01:14	06:09 ± 01:47	** < 0.0001**
Complete LGE module (min)	06:01 ± 02:28	09:21 ± 02:34	** < 0.0001**
LGE phase swap done	43 (22)	58 (29)	** < 0.001**

Data reported as mean ± standard deviation, counts (%), or as median (inter-quartile range Q1–Q3). Other abbreviations as in Tables 1 and 2

Significant *P* values highlighted in bold

*MI* myocardial infarction, *MVO* microvascular obstruction, *RV* right ventricle, *SAX* short axis

### Image quality

Artifacts encountered are presented in Fig. 1. Image quality was better for MOCO-LGE than bh-LGE (median [Q1–Q3] 0, 0–0 vs. 2, 0–3, *P* < 0.0001) even when limiting the analysis to clinically complex patients (0 [0–1] vs. 2 [1–4] *P* < 0.0001). Excellent image quality (score = 0) was achieved in 78% of patients imaged by MOCO-LGE compared to 27% by bh-LGE (*P* < 0.0001).

### LGE diagnoses

When reporting MOCO-LGE compared to bh-LGE images, blinded readers were more concordant in their rulings for presence/absence of LGE (bh-LGE vs MOCO-LGE kappa: 0.76, confidence interval [CI] 0.66–0.86 vs. 0.82, CI 0.74–0.91) and for LGE pattern (0.84, CI 0.75–0.92 vs. 0.87, CI 0.80–0.95 respectively). MOCO-LGE-based rulings retained greater concordance even when considering clinically complex patients only (bh-LGE vs. MOCO-LGE kappa 0.73, CI 0.61–0.85 vs. 0.83, CI 0.64–1.00 for presence/absence of LGE, and 0.80, CI 0.69–0.92 vs. 0.90, CI 0.78–1.02 LGE pattern).

### Diagnostic confidence

Diagnostic confidence was consistently greater for MOCO-LGE than for bh-LGE (higher score) irrespective of analytic method: basic analytic, 24.0 ± 16.2 vs. 15.9 ± 18.4 respectively, *P* < 0.0001; retained diagnostic, 22.8 ± 15.4 vs. 15.9 ± 16.9 respectively, *P* < 0.0001; and “Omary” correction, 32.2 ± 21.3 vs. 24. 5 ± 22.3 respectively, *P* < 0.0004).

### Scan time

The SAX LGE stack took nearly half the time by MOCO-LGE as it did by bh-LGE (3:22 vs. 6:09 min, *P* < 0.0001). Factors contributing to longer scan times by bh-LGE included the mandatory operator-defined pauses between successive breath-holds (each c. 5 s in duration) and the greater frequency of phase swaps/duplicate LGE imaging on account of suboptimal imaging (Table 2).

## Discussion

This real-world independent validation CMR study investigated whether a recently introduced ‘third generation’ LGE technique (PSIR free-breathing MOCO-LGE) delivered high image quality and faster scan times than ‘second generation’ PSIR bh-LGE. In a cohort of 400 consecutive patients referred to our center for clinical CMR just after introducing the MOCO-LGE sequence and with elementary staff training in its use, MOCO-LGE delivered better image quality, greater diagnostic confidence and faster scan times compared to traditional segmented bh-LGE. These findings held true even in the sickest of patients, such as those with dyspnea, arrhythmia, and multi-morbidity. Consequently, our centre (*n* = 10,000 scans/year) has now switched entirely to MOCO-LGE.

The comprehensive study by Piehler et al. [2] was the first to report on the unequivocal superiority of MOCO-LGE over bh-LGE, and similar albeit more preliminary data then followed for 3 T [15]. Piehler’s was a single-center, single-magnet study where each patient was imaged first by bh- and then by MOCO-LGE. Authors showed how acquisition time, the number of successfully scanned patients, and subjective image quality and diagnostic confidence by MOCO-LGE trumped those by bh-LGE. The present work validates the findings by Piehler and colleagues, using two magnets (both 1.5 T Aera) in a real-world setting where each patient was arbitrarily assigned to one or other technique by the scanning radiographer who was blinded to clinical complexity scores. We quantitatively appraised patient clinical complexity, image quality [16] and diagnostic confidence for objective classifications. Like Piehler et al., MOCO-LGE image quality was better than bh-LGE, even in complex patients, translating into increased diagnostic confidence for the reporting clinicians. Scanning was also faster using MOCO-LGE than with bh-LGE, and this was in spite of the sequence having only just been locally installed with relative staff inexperience consequently. The lack of bhs frees up the patient (who may be frail or tired by the end of the scan), and it also frees up the technologist increasing scan efficiency. The technologist may easily use idle time during the SAX acquisition to prescribe the long axes and apply them as three more free-breathing acquisitions. It also potentially simplifies the clinical workflow by giving technologists more time to think about the next patient to be scanned, increasing overall situational awareness of how the clinical list is operating. For MOCO-LGE applied to long-axis images, it is important to note that diaphragmatic motion here may lead to more through-plane motion which would be difficult for MOCO to correct. The SAX orientation, which mainly associates with in-plane translational motion lends itself better to adjustment by MOCO.

Time saved with MOCO-LGE and the cleaner images that result could translate into cost savings for health care systems by shortening the overall scan time per patient. Alternatively, time saved could be re-invested into acquiring additional clinically-indicated sequences to help better characterize a complex lesion or incidental finding thus impacting patient care.

To date, MOCO-LGE is still not ubiquitously available across CMR platforms and centers, so we urge manufacturers to swiftly invest in its distribution. Thanks to emerging joint motion feature learning approaches [17], it is now possible to envisage a future where routine scar identification takes place without the need for contrast. In the interim we shall need to carry on undertaking LGE imaging of the highest-possible quality to guide patient care. Our data adds to a compelling body of evidence which suggests that MOCO-LGE should replace bh-LGE as the scar imaging technique of choice for routine clinical care.

Limitations of the study include that the data presented is single-center and from a single manufacturer (Siemens), however we enrolled consecutive patients on two magnets and ensured a large enough sample size to mitigate some of these biases. The data we present for MOCO-LGE reflects the early transition period, immediately after sequence installation locally, so it is plausible (and indeed likely) that scan timings and image quality will have continued to improve as operator experience matured. Whilst analysis of all data by readers was blinded to patient data and the LGE sequence allocation, we clarify that bh and MOCO-LGE images by their very nature, have rather distinctive appearances, that limits the extent of blinding, even in the absence of other information.

## Conclusion

Just after its introduction to a high-throughput tertiary cardiac centre and after elementary staff training in its use, MOCO-LGE almost halves the time for LGE imaging compared to bh-LGE and improves diagnostic performance with high quality images and better reader confidence, even in the sickest of patients.

## Abbreviations

AF: Atrial fibrillation
bh: Breath-held
CMR: Cardiac magnetic resonance
DICOM: Digital imaging and communications in medicine
ECG: Electrocardiography
FISP: Fast imaging with steady state precession
FLASH: Fast low-angle shot
FOV: Field of view
GRAPPA: Generalized autocalibrating partial parallel acquisition
GRE: Gradient echo
IR: Inversion recovery
LGE: Late gadolinium enhancement
LV: Left ventricle
MOCO: Motion corrected
PD: Proton density
PSIR: Phase sensitive inversion recovery
SAX: Short axis
SSFP: Steady-state free precession
TE: Echo time
TI: Inversion time
TR: Repetition time

## References

[1] Kellman P, Arai AE (2012). Cardiac imaging techniques for physicians: late enhancement. J Magn Reson Imaging.

[2] Piehler KM, Wong TC, Puntil KS (2013). Free-breathing, motion-corrected late gadolinium enhancement is robust and extends risk stratification to vulnerable patients. Circ Cardiovasc Imaging.

[3] Kim RJ, Shah DJ, Judd RM (2003). How we perform delayed enhancement imaging. J Cardiovasc Magn Reson.

[4] Kellman P, Arai AE, McVeigh ER, Aletras AH (2002). Phase-sensitive inversion recovery for detecting myocardial infarction using gadolinium-delayed hyperenhancement. Magn Reson Med.

[5] Huber A, Hayes C, Spannagl B (2007). Phase-sensitive inversion recovery single-shot balanced steady-state free precession for detection of myocardial infarction during a single breathhold. Acad Radiol.

[6] Chung Y, Vargas J, Simonetti O, Kim RJR (2002). Infarct imaging in a single heart beat. J Cardiovasc Magn Reson.

[7] Kellman P, Larson AC, Hsu LY (2005). Motion-corrected free-breathing delayed enhancement imaging of myocardial infarction. Magn Reson Med.

[8] Ledesma-Carbayo MJ, Kellman P, Arai AE, McVeigh ER (2007). Motion corrected free-breathing delayed-enhancement imaging of myocardial infarction using nonrigid registration. J Magn Reson Imaging.

[9] Kellman P, Xue H, Hansen MS (2017). Free-breathing late enhancement imaging: phase sensitive inversion recovery (PSIR) with respiratory motion corrected (MOCO) averaging. Magnetom FLASH.

[10] Kramer CM, Barkhausen J, Flamm SD, Kim RJ, Nagel E (2008). Society for Cardiovascular Magnetic Resonance Board of Trustees Task Force on Standardized Protocols: standardized cardiovascular magnetic resonance imaging (CMR) protocols, Society for Cardiovascular Magnetic Resonance: Board of Trustees Task Force on Standardized Protocols. J Cardiovasc Magn Reson.

[11] Nordin S, Kozor R, Baig S (2018). Cardiac phenotype of prehypertrophic Fabry disease. Circ Cardiovasc Imaging.

[12] Xue H, Serensen T, Kellman P, Hansen M (2015). Distributed MRI reconstruction using Gadgetron-based cloud computing. Magn Reson Med.

[13] Klinke V, Muzzarelli S, Lauriers N (2013). Quality assessment of cardiovascular magnetic resonance in the setting of the European CMR Registry: description and validation of standardized criteria. J Cardiovasc Magn Reson.

[14] Ng CS, Palmer CR (2010). Analysis of diagnostic confidence: application to data from a prospective randomized controlled trial of CT for acute abdominal pain. Acta Radiol.

[15] Lin L, Wang Y, Cao J, Kong L, An J, Zhang T (2015). 3.0 T motion-corrected single-shot phase sensitive inversion recovery (PSIR) late gadolinium enhancement (LGE) in free-breathing patients compared with conventional segmented breath-held LGE. J Cardiovasc Magn Reson.

[16] Sievers B, Rehwald WG, Albert TSE (2008). Respiratory motion and cardiac arrhythmia effects on diagnostic accuracy of myocardial delayed-enhanced MR imaging in canines. Radiology.

[17] Xu C, Xu L, Gao Z (2018). Direct delineation of myocardial infarction without contrast agents using a joint motion feature learning architecture. Med Image Anal..

